# Supplementary material for: Splice-Junction-Based Mapping of Alternative Isoforms in the Human Proteome
Source: Cell Rep. Author manuscript; Available in PMC 2020 Jan 15. (PMC6961840; doi:10.1016/j.celrep.2019.11.026)
Supplement: 2 [file NIHMS1546469-supplement-2.zip › DF1/PXD010154/Thyroid/Thyroid_14_TG_NSDMGSRPESMGCR.pdf]

A

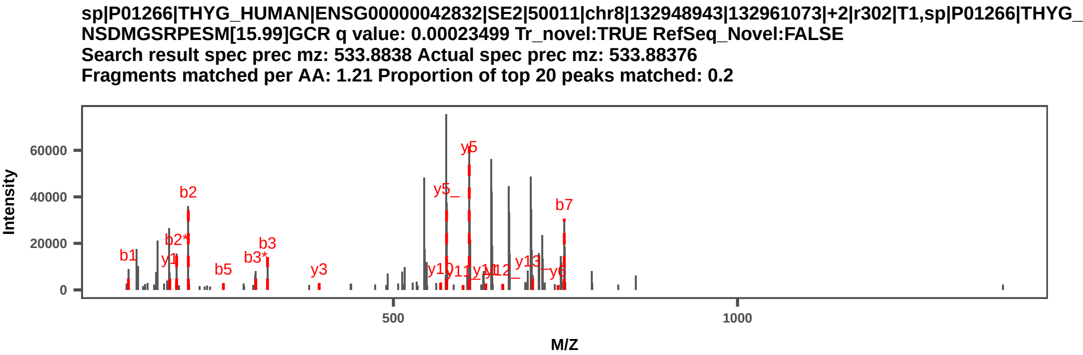
sp|P01266|THYG_HUMAN|ENSG00000042832|SE2|50011|chr8|132948943|132961073|+2|r302|T1,sp|P01266|THYG_
NSDMGSRPESM[15.99]GCR q value: 0.00023499 Tr_novel:TRUE RefSeq_Novel:FALSE
Search result spec prec mz: 533.8838 Actual spec prec mz: 533.88376
Fragments matched per AA: 1.21 Proportion of top 20 peaks matched: 0.2
Intensity
60000
40000
20000
0
b1
y1
b2*
b2
b5
b3*
b3
y3
y5_
y5
y10
y11
y12
y13
y6
b7
500
1000
M/Z

B

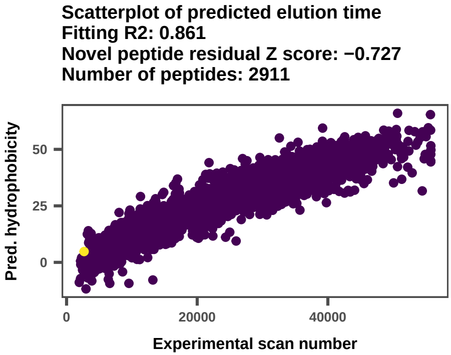
Scatterplot of predicted elution time
Fitting R2: 0.861
Novel peptide residual Z score: −0.727
Number of peptides: 2911
Pred. hydrophobicity
50
25
0
0
20000
40000
Experimental scan number

C

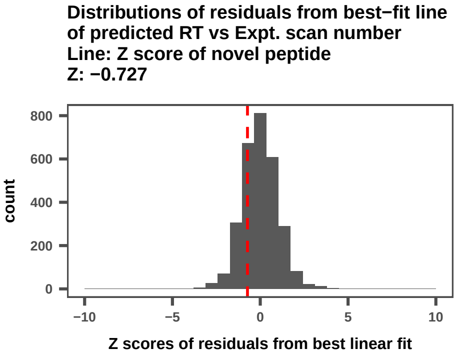
Distributions of residuals from best−fit line
of predicted RT vs Expt. scan number
Line: Z score of novel peptide
Z: −0.727
count
800
600
400
200
0
−10
−5
0
5
10
Z scores of residuals from best linear fit
